# Supplementary material for: Development of Nervilia fordii Extract-Loaded Electrospun PVA/PVP Nanocomposite for Antioxidant Packaging
Source: Foods. 2021 Jul 27;10(8):1728. doi: 10.3390/foods10081728 (PMC8391884; doi:10.3390/foods10081728)
Supplement: Supplementary file 1 [file foods-10-01728-s001.zip › foods-1295078-supplementary.pdf]

Supporting information

Table S1. Factors and levels in BBD

Factors	Symbols	levels		
		-1	0	1
Ethanol concentration (%)	A	55	60	65
Temperature (℃)	B	65	70	75
Solid-liquid rate	C	5:1	10:1	15:1

Table S2. Analysis of variance (ANOVA) for the experimental results of BBD

Source	Sum of Squares	df	Variance	F-Value	*p*-Value	Significance
Model	55.78	9	6.20	244.4	<0.0001	Significant
A	4.48	1	4.48	176.56	<0.0001	
B	18.61	1	18.61	733.87	<0.0001	
C	22.80	1	22.8	898.98	<0.0001	
AB	0.25	1	0.25	9.78	0.0167	
AC	0.3	1	0.3	11.99	0.0105	
BC	0.39	1	0.39	15.26	0.0059	
A^2	0.78	1	0.78	30.72	0.0009	
B^2	2.8	1	2.8	110.25	<0.0001	
C^2	4.56	1	4.56	179.79	<0.0001	
Residual	0.18	7	0.025			
Lack of fit	0.13	3	0.043	3.46	0.1309	Not significant
Pure error	0.049	4	0.012			
Total deviation	55.96	16				
	R^2 = 0.9968		R^2adj = 0.9927			

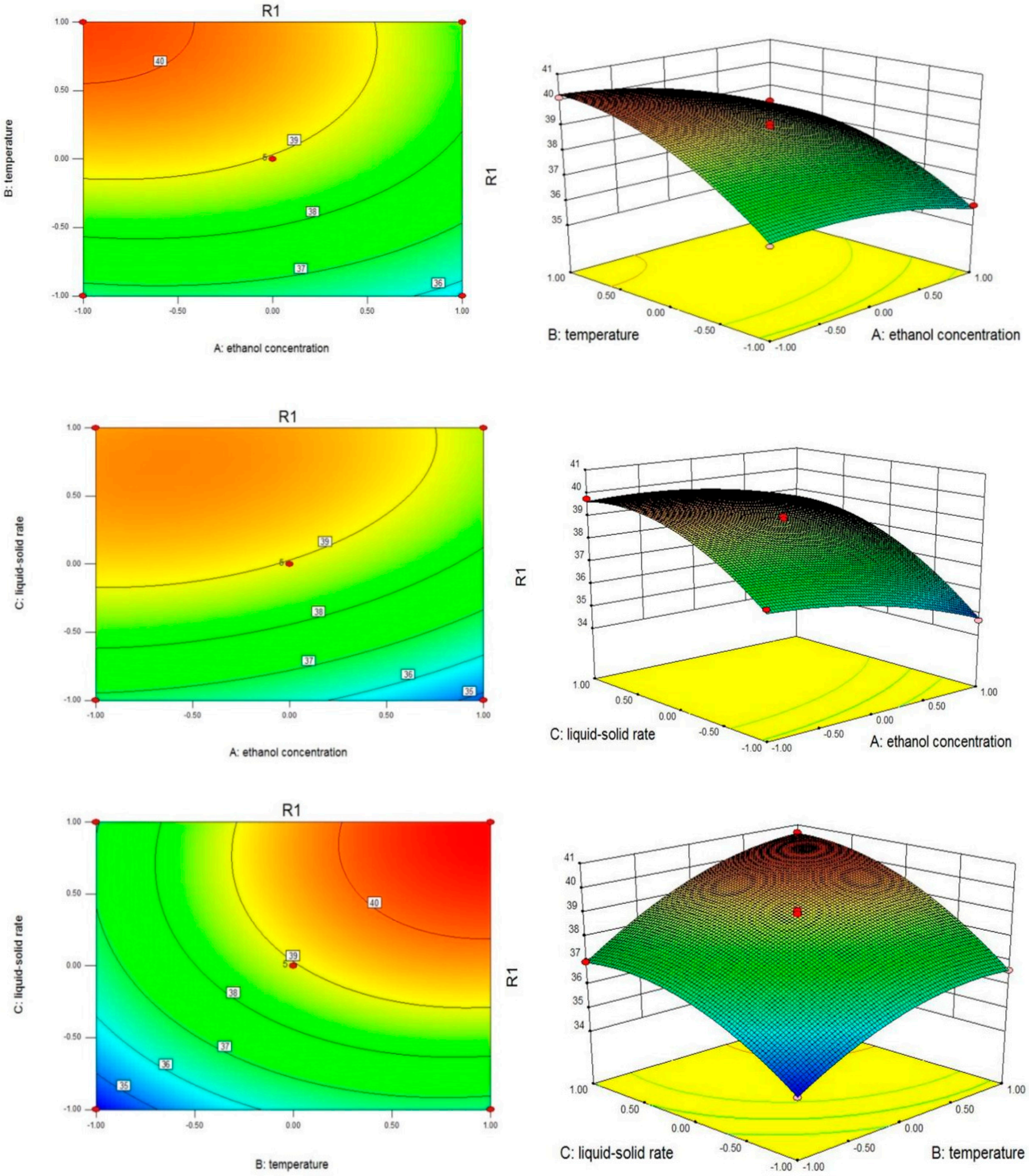

Figure S1. Response surface and corresponding contour plots of three factors on the DPPH radical scavenging activity
